# Effect of Nitrous Oxide During Oocyte Retrieval on In Vitro Fertilization Outcomes: A Retrospective Study

**DOI:** 10.7759/cureus.89146

**Published:** 2025-07-31

**Authors:** Shailendra Kumar, Harshini Medikondu, Dhruv Jain, Sainath Veeranki, Sana Y Hussain, Lokesh Kashyap, Reeta Mahey

**Affiliations:** 1 Anaesthesiology, Pain Medicine and Critical Care, All India Institute of Medical Sciences, New Delhi, New Delhi, IND; 2 Obstetrics and Gynaecology, All India Institute of Medical Sciences, New Delhi, New Delhi, IND

**Keywords:** anesthesia, in vitro fertilization, isoflurane, nitrous oxide, pregnancy

## Abstract

Background

Nitrous oxide (N₂O) is commonly used during general anesthesia for ovum pickup during in vitro fertilization (IVF) cycles. N₂O deactivates methionine synthetase, thereby reducing the amount of thymidine available for DNA synthesis in dividing cells, which might be the reason for the low implantation rate or increased frequency of early pregnancy loss. The aim of this study is to find out the IVF outcomes after exposure to either isoflurane or a combination of isoflurane + N₂O during anesthesia administration in the oocyte retrieval procedure.

Methods

This retrospective analysis was conducted on 250 patients undergoing IVF procedures who received anesthesia with isoflurane + N₂O or only isoflurane. Medical records were obtained for patient data, anesthesia details, and IVF outcomes. The primary objective was to compare the pregnancy rates between the two groups. The secondary objectives included comparing fertilization rates, oocyte grades, and cleavage rates.

Results

The pregnancy rates and cleavage rates measured on Day 2 and Day 3 of fertilization were similar in both groups. The fertilization rate (median(IQR)) was higher in the Iso group, but the difference was not statistically significant (air - 100 (87.5-100) vs. N₂O - 100 (83.3-100); P = 0.07). The number of oocytes retrieved of Grade 1 and Grade 2 quality was similar in both groups, while Grade 3 oocytes retrieved were significantly higher in the N_2_0 group.

Conclusion

While the use of N₂O during oocyte retrieval appears to be associated with an increased number of lower-quality oocytes, it does not negatively affect fertilization, cleavage, or pregnancy rates.

## Introduction

In vitro fertilization (IVF) is the most common method of assisted reproductive techniques (ART) for patients suffering from infertility. It involves the fusion of sperm and egg outside the female body and the transfer of the embryo into the female uterus. It consists of five steps: ovarian stimulation, follicular aspiration (egg collection), fertilization, embryo culture, and embryo transfer [1]. Transvaginal oocyte retrieval is accomplished by a minor surgical procedure, which involves the administration of anesthesia.

Anesthesia techniques involve the administration of intravenous agents, such as propofol and opioids, and inhalational agents, including isoflurane, sevoflurane, desflurane, and nitrous oxide (N₂O). The toxic effects of these agents may result in poor quality of oocytes and reduced fertility rates. General anesthetic agents penetrate quickly into the follicular fluid and, theoretically, may affect the fertilization process and cell cleavage through their mechanism of action at the cellular level [1-3]. Isoflurane is commonly employed for the maintenance of anesthesia during oocyte retrieval, and N₂O may be used along with it to reduce its requirement. N₂O deactivates methionine synthetase, thereby reducing the amount of thymidine available for DNA synthesis in dividing cells [4]. This inhibition, which lasts 24-72 hours, might be the reason for the low implantation rate or the increased frequency of early pregnancy loss. We hypothesized that exposure to N₂O during ovum pickup for IVF would lead to decreased pregnancy and fertilization rates.

The aim of this study is to find out the IVF outcomes after exposure to either isoflurane or a combination of isoflurane + N₂O during anesthesia administration in the oocyte retrieval procedure. The primary objective was to compare the pregnancy rates between the two groups. The secondary objectives included comparing fertilization rates, oocyte grades, and cleavage rates.

This study was previously presented as a scientific paper at the World Critical Care and Anaesthesiology Conference 2024, held in Bangkok on March 9, 2024.

## Materials and methods

This was a retrospective study conducted in the Department of Anaesthesiology, Pain Medicine & Critical Care and the Department of Obstetrics and Gynaecology of a tertiary care institute. Medical records of patients undergoing IVF procedures under anesthesia during five years (December 2018 to December 2023) were obtained after ethical clearance from the Medical Records Department. The records fulfilling the inclusion and exclusion criteria were analyzed further for the study.

Inclusion and exclusion criteria

Patients undergoing oocyte retrieval under general anesthesia with either isoflurane + air + oxygen or isoflurane + N₂O + oxygen as inhalational agents and those with American Society of Anesthesiologists (ASA) Grade 1 or 2 were included. Patients with ASA Grade 3 or 4 status, patients receiving total intravenous anesthesia, and procedures performed under sedation were excluded.

The following information was extracted from medical files: age/sex, weight, height, co-morbidities, type of infertility (primary/secondary), male or female factor for infertility, quality of oocyte (grade of oocyte), fertilization rate, cleavage rate, and pregnancy rate. The anesthesia details, including duration, type, and concentration of the inhalational agent/N₂O used, as well as any complications/events during anesthesia, were also noted.

IVF protocol

Ovarian stimulation was done with gonadotropins, recombinant follicle-stimulating hormone (Gonal F, Merck Serono, Geneva, Switzerland) and recombinant luteinizing hormone (Luveris, Merck Serono), and/or injection of human menopausal gonadotropin (HUMOG, Bharat Serum, Navi Mumbai, India), the dosages of which were individualized based on the patient's profile, including age, body mass index (BMI), anti-Müllerian hormone (AMH), antral follicle count (AFC), and ovarian response in previous cycles. Gonadotropin-releasing hormone antagonist (Inj Cetrorelix) 0.25 mg was added on Day 6 of stimulation and continued until the day of the trigger. The dose of the gonadotropins was adjusted based on the response. Final oocyte maturation was triggered with the injection of leuprolide 2 mg subcutaneously or recombinant human chorionic gonadotropin 250 mcg subcutaneously. Oocyte retrieval was performed 36-37 hours after the trigger. Following oocyte retrieval, cumulus oocyte complexes (COCs) were observed for nuclear and cytoplasmic abnormalities. Oocyte grading was done according to the number of cumulus cell layers: Grade 1: COC with five or more layers of cumulus cells; Grade 2: COC with three to four layers of cumulus cells; and Grade 3: COC with less than two cumulus cell layers [5]. Freshly ejaculated sperm samples were prepared by double-density gradient centrifugation, and conventional IVF or intracytoplasmic sperm injection (ICSI) was done, depending on the semen parameters.

Fertilization was assessed 18 hours after insemination, and the number/percentage of oocytes fertilized was noted. Furthermore, embryos were evaluated for cleavage on Days 2 and 3. Embryos with good grading were taken to Day 5 and assessed for blastulation. On Day 2/3 and Day 5 of development, embryos were evaluated and classified according to the recommendations of the Istanbul Consensus workshop [6]. All embryos from follicular stimulation and luteal stimulation were cryopreserved. Single frozen embryo transfer was performed in the next cycle or, depending on the patient's choice, within three months after endometrial preparation. All transfers were done under ultrasound guidance. Luteal phase support was maintained until 12 weeks of gestation or until the day of the pregnancy test, if the result was negative. Clinical pregnancy rates were calculated as per ultrasonographic visualization of one or more gestational sacs or fetal heartbeats. The clinical pregnancy rate included ectopic pregnancy as well.

Anesthesia protocol

For patients undergoing general anesthesia, they were taken to the operating theater. Intravenous access was obtained, and routine monitors were attached. Induction of anesthesia was performed by injection of fentanyl (2 mcg/kg) and propofol 1.5-2 mg/kg in titrated doses. The airway was secured using a laryngeal mask airway (LMA) device, and the patient was placed on mechanical ventilation. Anesthesia was maintained throughout the procedure using an isoflurane + N₂O + oxygen or isoflurane + air + oxygen mixture, with an inspiratory oxygen concentration of 40-50% and a minimum alveolar concentration (MAC) of 0.8-1. If N₂O was used, it was given at a concentration of 50%. After oocyte retrieval, anesthesia gases were discontinued, and the LMA was removed after the patient demonstrated spontaneous breathing efforts.

Statistical analysis

For sample size calculation, we took the primary outcome as the clinical pregnancy rate. Considering an average rate of 40%, we anticipated a 15% reduction in the pregnancy rate in the N₂O group. At a power of 80% and an alpha error of 5%, we would require 120 patients per group. We retrospectively included 125 patients in each group. All data were entered into Microsoft Excel (Microsoft Corporation, Redmond, Washington) and tabulated into the Air or N₂O group, depending on the agent received, for further analysis. The normality of the data was checked using the Kolmogorov-Smirnov test. The comparison of the variables, which were qualitative in nature, was analyzed using the chi-square test. If any cell had an expected value of less than 5, then Fisher's exact test was used. For quantitative non-parametric data, the Mann-Whitney U test was performed for comparison between two groups. If there was a significant difference in the baseline parameters, which could have affected the primary outcome, logistic regression was performed for categorical outcomes, and linear regression with robust standard errors was used for continuous outcomes to adjust for covariates. For statistical significance, a p-value of less than 0.05 was considered statistically significant.

## Results

Data were retrospectively collected until 125 patients were included in both groups. The time period for these data was December 2018 to December 2023. In the Air group, two patients had diabetes and three patients had hypertension, while one patient had diabetes and one had hypertension in the N₂O group. Additionally, one patient had a history of abdominal tuberculosis in the Air group. None of the patients were smokers or alcoholics. The baseline characteristics of patients are shown in Table 1.

**Table 1:** Demographic and baseline characteristics *statistically significant. Continuous data presented as median (interquartile range) n = number of patients; ASA = American Society of Anesthesiologists; BMI = body mass index

Variables	Air (n=125)	N_2_O (n=125)	P-value
Age (years)	30 (28–34)	32 (30–35)	0.007*
BMI (kg/m^2^)	23.8 (22.2–26.2)	24.2 (22.4–26.6)	0.36
ASA grading I/II (n)	107/18	99/26	0.18
Hypothyroidism (n)	9	22	0.01*
Obesity (n)	2	3	1
Duration of procedure (minutes)	45 (40–60)	45 (45–60)	0.15
Type of infertility (1°/2°)	102/23	106/19	0.5

Oocytes of four patients in the Air group and two patients in the N₂O group did not proceed for fertilization. Therefore, the fertilization rate, cleavage rate, and pregnancy rate were not analyzed for these patients, and the data are presented in Table 2. The fertilization rate was higher in the Air group, but the difference was not statistically significant. The cleavage rate measured on Days 2 and 3 of fertilization was similar in both groups. The pregnancy rate was also similar in both groups.

**Table 2:** Comparison of fertilization, cleavage, and pregnancy rates Continuous data presented as median (interquartile range) n = number of patients; CI = confidence interval

Parameters	Air (n=121)	N_2_O (n=123)	P-value
Fertilization rate	100 (87.5–100)	100 (83.3–100)	0.1
Cleavage rate			
Day 2	100 (100–100)	100 (100–100)	0.79
Day 3	80 (50–100)	66.6 (50–100)	0.45
Pregnancy rate n(%)	47 (38.8); 95% CI (30.1–48.1)	47 (38.2); 95% CI (29.6–47.4)	0.92

The number of oocytes retrieved of Grades 1 and 2 quality was similar in both groups, while Grade 3 oocytes retrieved were significantly higher in the N_2_0 group (P < 0.001, Table 3).

**Table 3:** Number of different grades of oocytes in each group Data presented as median (interquartile range) CI = confidence interval

Oocyte Grade	Air (n=125)	N_2_O (n=125)	P-value
Grade 1	2 (1–5); 95% CI (0.9–3.1)	2 (1-3); (95% CI 1.45–2.55)	0.64
Grade 2	4 (2–5); 95% CI (3.17–4.83)	3 (2–4); 95% CI (2.45–3.55)	0.07
Grade 3	1 (0–3); 95% CI (0.17–1.83)	3 (2–4); 95% CI (2.45–3.55)	< 0.001

An adjusted analysis was carried out to account for covariates, including hypothyroidism and age, as they were unequally distributed between the two groups. For the outcome of the pregnancy rate, logistic regression was performed. On adjustment, pregnancy rate was not found to be statistically significant (P = 0.95). For outcomes such as fertility rate, cleavage rate, and oocyte grading, linear regression analysis was performed. After adjusting for the factors, the Grade 3 oocytes were significantly higher in the N₂O group (P < 0.001), while the fertilization and cleavage rates were similar. Thus, after adjusting for covariates, the results remained unchanged.

## Discussion

This retrospective study aimed to evaluate the impact of N₂O use during oocyte retrieval on IVF outcomes by comparing two groups of patients undergoing general anesthesia with isoflurane and air versus isoflurane and N₂O. The primary outcome was the pregnancy rate, and secondary outcomes included oocyte grading, fertilization rates, and cleavage rates. The study offers valuable insights into the potential cellular and clinical effects of anesthetic agents on reproductive success.

One of the most prominent findings in this study was the significantly higher number of Grade 3 oocytes retrieved in the N₂O/Iso group compared to the Air/Iso group (P < 0.001). While Grade 1 and Grade 2 oocyte counts remained similar across groups, this increase in lower-quality oocytes raises concern regarding N₂O's impact on oocyte morphology and cytoplasmic integrity.

On a cellular level, N₂O has been shown to irreversibly inhibit methionine synthetase, an enzyme essential for DNA synthesis and methylation reactions, by oxidizing its cobalamin (vitamin B12)-dependent cofactor. This results in reduced thymidine availability, potentially affecting dividing cells such as oocytes and preimplantation embryos. Although this biochemical alteration has been well-demonstrated in animal studies and hepatocytes, its clinical relevance in the context of short-term N₂O exposure during oocyte retrieval remains uncertain due to the gas's low solubility and transient follicular exposure [7].

These findings align with observations by Gonen et al., who reported that general anesthesia with N₂O was associated with significantly lower clinical pregnancy and delivery rates compared to local or epidural techniques. Their study attributed these outcomes to possible effects on oocyte quality or endometrial receptivity [7]. Additionally, recent research by Tola [8] has also emphasized the potential sensitivity of gametes to anesthetic agents, showing that ketamine was associated with significantly reduced fertilization rates compared to propofol and ketamine-propofol combinations, even after adjusting for sperm motility. This highlights the broader concern that some anesthetics may impair cellular environments critical for fertilization, despite not always affecting pregnancy rates. The current study, while confirming the impact on oocyte grading, did not replicate the decline in pregnancy outcomes, possibly due to differences in exposure duration, adjunct medications, and methodological approaches.

Despite the poorer oocyte quality observed in the N₂O/Iso group, fertilization rates remained statistically comparable between the two groups (P = 0.1). This finding is consistent with those of Rosen et al., who conducted a randomized trial comparing isoflurane alone with isoflurane combined with N₂O, reporting no significant differences in fertilization or pregnancy rates [9]. Similarly, Hayes et al. found that while anesthesia length and order of oocyte retrieval influenced cleavage and fertilization to some extent, there was no strong evidence directly implicating anesthetic agents themselves [3].

The cleavage rates observed on Days 2 and 3 post-fertilization were also similar in both groups, indicating that early embryonic development was not adversely affected by the inclusion of N₂O in the anesthetic regimen. These results are reassuring, as they suggest that the presence of lower-grade oocytes did not translate into impaired developmental competence, at least during the preimplantation phase. Tola [8] also examined cleavage and embryo quality, finding no significant differences between various anesthesia protocols. This reinforces the conclusion that fertilization and early development may remain unaffected by anesthesia exposure, even in the presence of poorer-quality oocytes or reduced sperm motility.

No significant difference in pregnancy rates was noted between the two groups (P = 1.0), reinforcing the idea that short-term N₂O exposure during oocyte retrieval may not be harmful in terms of achieving pregnancy. This observation supports the findings from multiple studies, including those by Matsota et al. [1] and Sharma et al. [2], who reviewed the impact of various anesthesia techniques and concluded that there is insufficient evidence to discourage the use of N₂O solely based on theoretical risks. Interestingly, Tola [8] observed that while fertilization was affected by the type of anesthetic agent, implantation and clinical pregnancy rates were significantly lower only in cases where anesthesia duration exceeded 30 minutes​ [8]. This observation suggests that it may not only be the agent used but also the duration of exposure that influences downstream IVF outcomes, a crucial consideration that supports efforts to minimize procedural time during oocyte retrieval.

However, the clinical relevance of increased Grade 3 oocytes must be interpreted cautiously. While lower oocyte quality does not always prevent successful fertilization or implantation, it may be more consequential in patients with diminished ovarian reserve or advanced maternal age, where every retrieved oocyte is critical. In such populations, avoiding even minimal potential risks may be important.

Beyond the specific effect of N₂O, the broader literature has explored the impact of various anesthetic agents on IVF success. For example, Hadimioglu et al. found no significant difference in recovery characteristics or IVF parameters among patients sedated with propofol-fentanyl, propofol-alfentanil, or midazolam-based regimens [10]. In contrast, Herzberger et al. recently observed significantly lower fertilization rates in patients receiving general anesthesia with propofol compared to those undergoing oocyte retrieval without anesthesia, though pregnancy and live birth rates were unaffected [11].

In our study, the standardized use of isoflurane as the maintenance agent across both groups ensured that N₂O was the primary differentiating factor, thereby strengthening the validity of our findings.

Strengths and limitations

The strengths of this study include its large sample size, rigorous inclusion/exclusion criteria, and focused comparison between two standardized anesthetic regimens. The analysis of multiple IVF parameters (oocyte grade, fertilization, cleavage, and pregnancy) provides a comprehensive view of N₂O's potential impact.

The study's limitations include its retrospective nature, which may introduce bias in record selection and data completeness. In addition, although anesthetic protocols were generally consistent, variations in exact N₂O concentration, isoflurane dose, and procedure duration were not controlled prospectively. Fertilization and pregnancy rates also depend on various procedural and maternal factors: embryo quality, conventional or ICSI, baseline ovarian reserve, AMH levels, and history of polycystic ovarian syndrome or endometriosis. These factors were not analyzed and adjusted in our study, which could have affected the outcomes. Furthermore, long-term outcomes, such as the live birth rate and neonatal health, were not assessed.

Future directions

Prospective, randomized controlled trials are warranted to further clarify whether N₂O has a measurable impact on IVF outcomes, particularly in high-risk populations. Studies analyzing follicular fluid for oxidative stress or methylation changes could provide insight into how N₂O affects oocyte development at the cellular level. Additionally, evaluating repeated exposures to N₂O across multiple IVF cycles could be useful in shaping future anesthetic protocols. The findings from Tola's study [8] emphasize the importance of studying not only outcomes like fertilization and pregnancy but also intermediary steps, such as oocyte maturation and embryo grading, to understand the nuanced impact of anesthesia on reproductive potential.

## Conclusions

In summary, while N₂O use during oocyte retrieval appears to be associated with an increased number of lower-quality oocytes, it does not negatively affect fertilization, cleavage, or pregnancy rates. Nevertheless, in patients with limited ovarian reserve, careful consideration should be given to selecting the most appropriate anesthetic technique. Further prospective studies are necessary to confirm these findings and guide clinical decision-making.
